# Early Radiation-Induced Changes in Lung Tissue and Intercellular Junctions: Implications for Tissue Repair and Fibrosis

**DOI:** 10.3390/pathophysiology31040039

**Published:** 2024-09-24

**Authors:** Ekaterina S. Karetnikova, Alexandra A. Livanova, Arina A. Fedorova, Alexander G. Markov

**Affiliations:** 1 Department of General Physiology, St. Petersburg State University, 199034 St. Petersburg, Russia; 2 Interoception Laboratory, Pavlov Institute of Physiology RAS, 199034 St. Petersburg, Russia

**Keywords:** lung, epithelium, intercellular junction, tight junction, E-cadherin, claudin, occludin, ionizing radiation, apoptosis, tissue structure

## Abstract

Early changes in lung tissue following ionizing radiation (IR) initiate processes that may lead to either regeneration or fibrosis. Intercellular junction proteins play a crucial role in the organization and function of epithelial tissues, both under normal conditions and after injuries. Alterations in the expression and localization of these proteins can influence the fate of epithelial cells. This study aims to investigate the effects of IR on lung tissue structure, as well as on the levels and distribution of intercellular junction proteins. Wistar rats were subjected to total X-ray irradiation at doses of 2 and 10 Gy. Lung tissue samples were collected for Western blot and histological analysis 72 h post-IR. IR at doses of 2 and 10 Gy led to structural changes in lung tissue and elevated levels of E-cadherin. The 10 Gy IR resulted in increased claudin-4 and occludin in lung parenchyma, decreased claudin-8 and claudin-12 in bronchial epithelium and endothelium, and suppression of apoptosis. Data evaluation indicated that alterations in the protein composition of intercellular junctions are essential processes in lung tissue at early stages after IR, and at least some of these alterations are associated with adaptation.

## 1. Introduction

Damage to lung tissue during radiation therapy leads to the development of complications such as pneumonitis and/or lung fibrosis, the occurrence of which limits the intensity and effectiveness of antitumor treatment [1,2,3,4]. The peculiarity of post-radiation pathologic reactions is a long-term mostly asymptomatic steady progression leading to respiratory insufficiency [4,5,6]. Despite a large number of studies and available data on the mechanisms of development and regulation of post-radiation changes in the lungs, currently, the treatment and secondary prevention of lung fibrosis are ineffective [5,7,8]. Early after exposure to ionizing radiation (IR), subsequent pathological processes are triggered. Studies of the processes occurring in the lung tissue in the first few days after IR are promising, as the data obtained may show new ways of treatment to shift the balance towards lung tissue regeneration and prevent the development of post-radiation pulmonary fibrosis.

Direct and indirect damage of macromolecules by IR and reactive oxygen species triggers programmed cell death [9], synthesis, and activation of proinflammatory cytokines, which in turn initiate the processes of aseptic inflammation [7,10]. In addition to triggering aseptic inflammation, cytokines increase the activity of inducible nitric oxide synthase (iNOS) and stimulate the activity of dimethylarginine dimethylaminohydrolase 1 (DDAH1), an enzyme that cleaves asymmetric dimethylarginine, which is an iNOS inhibitor. Increased iNOS activity leads to further damage of lung tissue and intensification of aseptic inflammation, which induces the synthesis of transforming growth factor beta, triggering the processes of epithelial–mesenchymal transition (EMT), significant for the development of lung fibrosis [8]. The relationship between IR doses, exposure metrics, and the timing of the onset and progression of the aforementioned early pathological processes is not yet fully understood. Moreover, data from most studies were obtained several weeks or months after IR exposure when aseptic inflammation or lung fibrosis had already developed [7,11]. While it is the processes occurring in the first several days after IR that initiate subsequent pathologic changes, several researchers have addressed the problem of early damage in lung tissue [12,13]. Identification of key changes occurring in the first few days after IR may show ways to correct these changes in order to prevent initiation of further pathologic reactions and preserve lung function. Thus, assessment of early post-IR changes in lung tissue is an actual and promising area of research.

Alveolar and bronchial epithelium along with endothelium are the main structural and functional components of lung tissue. The structure, intercellular permeability and barrier properties of epithelial tissues are determined by multiprotein complexes of intercellular junctions, namely, adherent and tight junctions [14,15,16]. E-cadherin, which is part of adherent junctions, forms a zone of adhesion of cells to each other and contributes to the organization of epithelial layers [14,15]. Exposure to IR initiates EMT, with a decrease in the level of E-cadherin in lung tissue [17,18]. Tight junctions (TJs) are multiprotein complexes consisting of transmembrane proteins and associated cytoplasmic proteins. The transmembrane proteins include proteins of the claudin family, as well as occludin and tricellulin. Claudin family proteins are responsible for the pore pathway of epithelial intercellular permeability [16,19,20,21]. TJs bind to the actomyosin ring located in the cytoplasm. Changes in the tension of actomyosin rings have been shown to affect intercellular permeability. In turn, the tension of actomyosin rings is regulated by the myosin light chain kinase (MLCK) [22,23,24]. The levels and localization of TJ proteins have been shown to be changed under various pathogenic conditions. Moreover, various injuries in the first few days cause distinct changes in the protein composition of the TJ, leading to specific modifications in intercellular permeability and other functions of the epithelium [25,26,27,28,29,30]. In addition to the abovementioned functions, a number of adherent and TJ proteins have regulatory properties, affecting such processes as proliferation [31,32], differentiation [31,33], EMT [32,33], and apoptosis [34]. Thus, the protein composition and function of intercellular junctions are altered early after various lesions, and these changes, in turn, affect the fate of epithelial cells. However, so far, no one, to the best of our knowledge, has studied the effect of IR on TJs in lung tissue.

The aim of this study was to evaluate the early effects of total X-ray irradiation at doses of 2 and 10 Gy on the lung tissue structure, as well as on the level and distribution of intercellular junction proteins in rat lungs. Medium and high radiation doses were defined as doses below (2 Gy) and above (10 Gy) LD50/30 for the selected model [35]. Assessment of the influence of IR in doses of 2 and 10 Gy on the histological structure of lungs showed that 72 h after irradiation the integrity of epithelia was preserved, and there were changes in the structure of lung tissue in the form of foci of microdystelectasis and alveolar hyperinflation. An increase in E-cadherin levels was detected after IR at doses of both 2 and 10 Gy. The 10 Gy IR caused an increase in claudin-4 in the alveolar epithelium, a decrease in claudin -8 and -12 in the endothelium, and an abundance of occludin in lung tissue, as well as inhibition of apoptosis. Evaluation of the data revealed that at least some of the identified alterations are associated with adaptive processes in lung tissue.

## 2. Materials and Methods

### 2.1. Animals

Male Wistar rats (*Rattus norvegicus*) with body weight of 190–230 g were included in the study. Animals were housed in cages in a room with a regulated and controlled temperature (22 ± 2 °C) and humidity, with changing light/dark cycles every 12 h. Rats had access to water and food ad libitum. Body weight of the animals was measured every 24 h throughout the study period. All procedures were performed in accordance with the recommendations of the Guide for the Care and Use of Laboratory Animals [36]. The conduct of the study was fully compliant with the EU requirements set out in the Directive 2010/63/EU on animal experiments and was approved twice by the Ethical Committee for Animal Research of St. Petersburg State University on 13 December 2017 No 131-03-5 and 29 April 2024 No 131-03-10.

Rats were randomized to three groups. Animals of the first (*n* = 8), second (*n* = 8), and third (*n* = 8) groups were irradiated at doses of 0 Gy, 2 Gy, and 10 Gy, respectively. Whole-body X-ray irradiation was performed on an orthovoltage therapeutic X-ray unit RUM-17 (MosRentgen, Russia); dose rate—0.31 Gy/min; focal distance of the X-ray tube—50 cm. To restrict animal movement during the irradiation procedure, rats were placed in an aerated plexiglass box. The rats of the first group that received sham irradiation (0 Gy) were under the deactivated X-ray tube for 30 min. At 72 h after IR, the animals were injected intraperitoneally with tribromoethanol at a dose of 750 mg/kg body weight. Once the rats had achieved deep anesthesia, lung tissue samples were collected. The samples were fast frozen at −80 °C for Western blot studies or fixed with 10% buffered formalin (BioVitrum, St. Petersburg, Russia) for histologic studies.

### 2.2. Western Blot

Frozen lung samples were placed in mini tubes with chilled lysis buffer (150 mM NaCl, 10 mM Tris-HCl (pH 7.4), 0.5% Triton X-100, 0.1% SDS, Complete mini-EDTA-free protease inhibitors cocktail (Roche, Basel, Switzerland)), homogenized using a Retsch MM400 homogenizer (Retsch, Haan, Germany) and lysed for 20 min on ice with three times stirring of the samples. The samples were then centrifuged in a chilled centrifuge (Eppendorf, Hamburg, Germany) at 4 °C, 14,000× *g* for 15 min. The supernatants were collected and frozen at −80 °C. The total protein concentration in supernatants was measured on a SPECTROstar Nano spectrophotometric microplate reader (BMG Labtech, Ortenberg, Germany) using the Pierce Rapid Gold Bicinchoninic Acid Protein Assay Kit (Thermo Fisher Scientific, Waltham, MA, USA). Next, the samples were diluted with lysis buffer and Laemmli buffer (Bio Rad, Hercules, CA, USA) to equal total protein concentrations and heated for 10 min at 95 °C.

Electrophoresis of rat lung lysates was performed in a vertical electrophoresis chamber (Bio Rad, Hercules, CA, USA) in 12% polyacrylamide gel (Serva, Heidelberg, Germany); proteins were transferred from gels to polyvinylidene fluoride (PVDF) membranes (Bio Rad, Hercules, CA, USA) using a Hoefer Semi Dry Transfer Unit (Fisher Scientific, Waltham, MA, USA). Then, PVDF membranes were blocked using EveryBlot Blocking Buffer (Bio Rad, Hercules, CA, USA) for 5 min at 24 °C and membranes were incubated overnight at 4 °C in one of the solutions of primary rabbit antibodies to E-cadherin, claudin-18, MLCK, α-SMA (AF0131, DF9392, AF5314, AF1032, Affinity Biosciences, Changzhou, Jiangsu, China), to claudin-1, -2, -3, -4, -5, -8, -12, occludin, tricellulin, DDAH1 (#51-9000, #51-6100, #34-1700, #36-4800, #35-2500, #40-0700z, #38-8200, #71-1500, #48-8400, #PA5-52278, Thermo Fisher Scientific, Waltham, MA, USA), cleaved caspase-3 (9661, Cell Signalling, Danvers, MA, USA), or incubated in a solution of primary mouse antibodies to β-actin (ab8224, Abcam, Cambridge, UK) for 2 h at 24 °C. Next, the primary antibodies were washed out, and the membranes were incubated with one of the horseradish peroxidase-conjugated secondary antibodies to rabbit (AB205718, Abcam, Cambridge, UK) or to mouse (AB205719, Abcam, Cambridge, UK) for 1 h at 24 °C. Incubation of membranes in the solutions of primary or secondary antibodies was carried out under constant stirring. The secondary antibodies were washed out. Chemiluminescence images were acquired on a ChemiDoc XRS+ Imaging System (Bio-Rad, Hercules, CA, USA) using Clarity Western ECL solution (Bio-Rad, Hercules, CA, USA). Processing of the acquired bend images was performed with Image Lab 6.1 Software (Bio-Rad, Hercules, CA, USA). The chemiluminescence intensity of bends of the tested proteins was normalized to the chemiluminescence intensity of β-actin bends. The mean levels of the tested proteins in lung samples of sham-irradiated rats were taken as 100%.

### 2.3. Histological Analysis and Morphometry

Formalin-fixed rat lung samples were dehydrated in a series of alcohol solutions of increasing concentration, paraffinized, and embedded in paraffin. Histological sections 5 μm thick were sliced using a Leica RM2265 rotary microtome (Leica, Wetzlar, Germany) and placed on SuperFrost glass slides (Thermo Fisher Scientific, Waltham, MA, USA). The sections attached to the slides were then stained with haematoxylin and eosin (H&E). The stained sections of rat lungs were analyzed under a Leica DMI6000 light microscope (Leica, Wetzlar, Germany). Images of stained lung sections were obtained using a color digital CCD camera (Leica, Wetzlar, Germany).

Analysis and morphometry of the obtained images were performed in ImageJ 1.52p software (NIH, Madison, WI, USA). All measurements were performed on lung tissue images taken at 100× magnification. In order to make image processing uniform, an equal number of images from each animal group was combined into stacks (“Image -> Stacks -> Images to Stack”), and all subsequent image conversion steps were applied to the image stacks. To measure the length of air-tissue boundaries, in the first step, the areas occupied by lung tissue were separated from the air zones using the command “Process -> Binary -> Make Binary”. Then, to improve the separation of tissue regions, the command “Process -> Binary -> Dilate” was applied to the stacks of black-and-white images. In the next step, air- tissue boundaries were selected using the “Process -> Binary -> Outline” command. The “Analyse -> Measure” command allowed us to measure the extent of air-tissue boundaries on images of lung parenchyma (Figure S1). In order to measure the percentage of lung tissue area occupied by cell nuclei, the command “Image -> Colour -> Colour Deconvolution -> Colour Deconvolution -> Vectors: H&E” was applied to the image stacks. Three stacks of images were obtained from the image stack after this command was completed. The first stack contained images of nuclei stained with haematoxylin, the second stack contained images of tissue stained with eosin, and the third stack contained green staining that was noninformative in the selected variant of the study (the third stack with images was closed). The commands “Process -> Binary -> Make Binary” and “Analyse -> Measure” were sequentially applied to the first two stacks of images, which allowed us to measure the areas of lung tissue and nuclei in the micrographs (Figure S1). The data obtained allowed us to calculate the percentage of lung tissue area occupied by cell nuclei.

### 2.4. Immunofluorescent Staining

The sections attached to slides were deparaffinized and rehydrated. Heat-induced antigen retrieval was then performed in citrate buffer pH 6.0 (BioVitrum, Saint-Petersburg, Russia) at 100 °C for 20 min. Blocking of nonspecific lung tissue interaction with secondary antibodies was performed for 2 h at 24 °C with Normal Goat Blocking Buffer (Elabscience, Wuhan, China). Sections were incubated in one of the solutions of primary antibody to E-cadherin and claudin-18 (AF0131 and DF9392, respectively; Affinity Biosciences, Changzhou, China), to claudin-1, -3, -4, -5, -8, -12, and occludin (#51-9000, #34-1700, #36-4800, #35-2500, #40-0700z, #38-8200, and #71-1500, respectively; Thermo Fisher Scientific, Waltham, MA, USA) overnight at 4 °C in a humidified chamber. The primary antibodies were then washed out and the slices were incubated in Alexa Fluor Plus 488 (a32731, Thermo Fisher Scientific, Waltham, MA, USA) Goat anti-rabbit Secondary Antibody solution for 2 h at 24 °C. Finally, the secondary antibodies were washed out, the slices were mounted under coverslips with glycerol containing DAPI, and the coverslips were fixed with nail polish. Stained sections were analyzed and imaged using a Leica TCS SP5 MP fluorescence confocal scanning microscope (Leica, Wetzlar, Germany). The images were processed using Image Lab 6.1 software (Bio-Rad, Hercules, CA, USA) and ImageJ software (NIH, Madison, WI, USA).

### 2.5. Statistics

Statistical analysis of data was performed in Microsoft Excel 2016 (Microsoft Corp., Redmond, WA, USA) and GraphPad Prism 8 software (GraphPad, San Diego, CA, USA). Sample sizes were calculated based on data from the Western blot pilot study, with the probability of a first-type error taken as 0.05 and the probability of a second-type error as 0.2. The results of calculations showed that the required and sufficient sample size was 8 animals in each group. After the data from the main study were obtained, all data groups were checked for outliers using the ROUT (Q = 1%) and Grubbs’ (α = 0.05) tests, and identified outliers were excluded. The significance of differences between groups was assessed using one-way ANOVA followed by Tukey’s multiple comparisons test.

Lung samples from four animals (*n* = 4) were randomly selected from each study group for lung tissue morphometric studies. Fifteen random fields of view (*N* = 15) of the lung parenchyma were imaged for each of the samples taken in the study. Therefore, 60 (*N* = 60) images of lung parenchyma were taken from each studied group for morphometric analysis. Statistical processing of the data obtained during morphometric analysis was performed using nested one-way ANOVA followed by Tukey’s multiple comparisons test. All data are presented as mean ± standard error of the mean.

## 3. Results

### 3.1. IR Affects Body Weight in Rats

**Figure 1:**
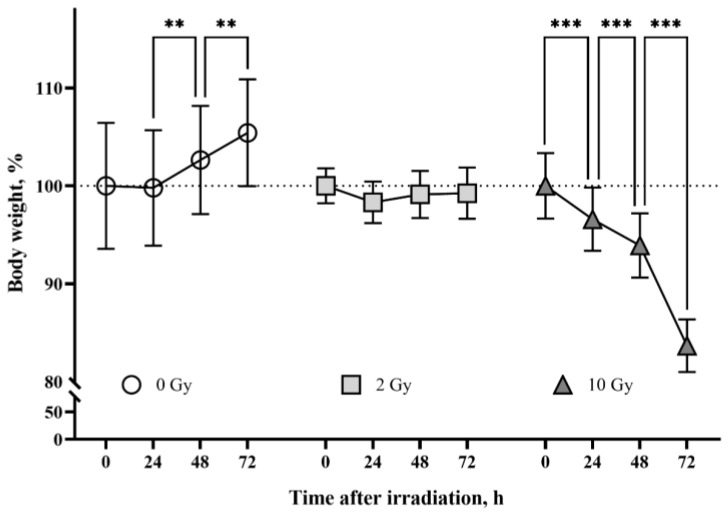
The effects of ionizing radiation (IR) on body weight in rats. The group of sham-irradiated rats had an increase in mean body weight (*n* = 8). IR 2 Gy caused arrest of body weight gain in animals (*n* = 8). The group of rats irradiated at the dose of 10 Gy had a daily decrease in body weight (*n* = 8). Data are presented as a percentage of the average weight of the groups at the beginning of the tests. Two-way ANOVA followed by Tukey’s multiple comparisons test, ** *p* < 0.01, *** *p* < 0.001.

The body weight of rats from the sham-irradiated control group increased throughout the observation period (*p* < 0.01) (Figure 1). The group of animals irradiated with 2 Gy had an arrest of weight gain. The mean body weight of 10 Gy IR rats decreased by 16% from the mean body weight on the day of irradiation. The detected changes in rat body weight are consistent with previously published data [37,38,39] and confirm the effect of IR doses.

### 3.2. Early Changes in Lung Tissue after IR

**Figure 2:**
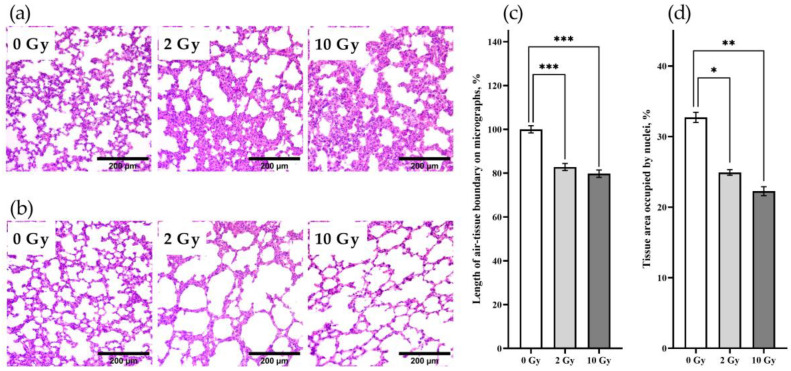
IR at doses of 2 and 10 Gy causes changes in the structure of lung tissue 72 h after exposure. Rat lung tissue stained with H&E (**a**,**b**). IR at doses of 2 and 10 Gy causes microdystelectasis (**a**), foci of alveolar hyperinflation (**b**), a decrease in the extent of the air–tissue boundary (**c**), and a decrease in the percentage of lung tissue area occupied by nuclei (**d**). Nested one-way ANOVA followed by Tukey’s multiple comparisons test, * *p* < 0.05, ** *p* < 0.01, *** *p* < 0.001; *N* = 60, *n* = 4.

Hematoxylin and eosin (H&E)-stained lung sections of rats of the studied groups were analyzed. The lung tissue of sham-irradiated animals had normal morphological structure. IR at doses of 2 and 10 Gy caused mosaic restructuring of lung tissue, represented by the appearance of foci of microdystelectasis (Figure 2a) and alveolar hyperinflation (Figure 2b). The absence of desquamated cells in the air spaces of alveoli after 2 Gy and 10 Gy IR indicates preservation of alveolar epithelium integrity on the third day after irradiation. In order to assess the impact of the identified structural alterations in lung tissue on the aerohematic barrier, we measured the length of air–tissue boundaries on lung tissue slices. A decrease in the length of air–tissue boundaries in the lungs of rats irradiated at doses of 2 and 10 Gy was detected (Figure 2c), probably reflecting a proportional decrease in the aerohematic barrier area and lung diffusion capacity. In order to evaluate the effect of IR on the interstitium, the percentage of lung tissue area occupied by cell nuclei was measured (Figure 2d). The area of nuclei was 33 ± 0.7% of the total lung tissue area in the control group. IR at doses of 2 and 10 Gy caused a decrease in the area of nuclei in lung tissue to 25 ± 0.4% (*p* < 0.05) and 22 ± 0.6% (*p* < 0.01), respectively. Thus, the lungs at the histological level are sensitive to IR at the dose of 2 and more Gy; herewith, structural tissue changes on the third day after IR occur against the background of preservation of alveolar epithelium integrity.

**Figure 3:**
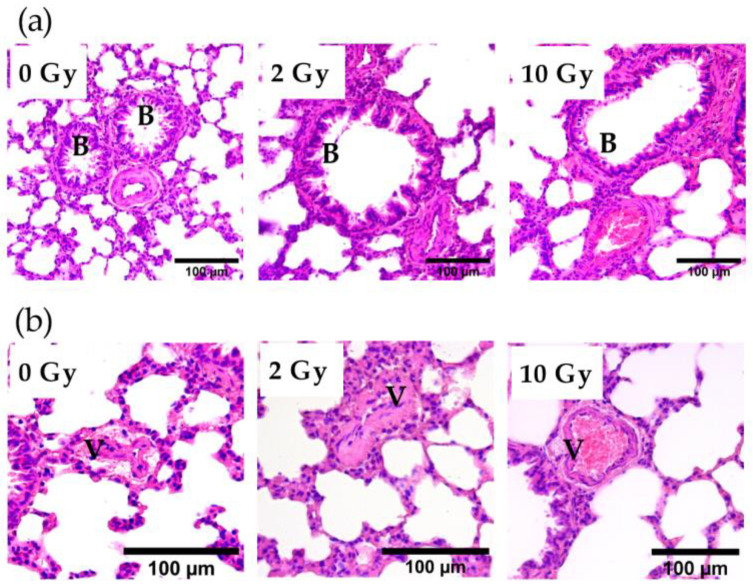
Bronchioles (**a**) and vessels (**b**) in the lung tissue of rats irradiated at doses of 0, 2, and 10 Gy. The 10 Gy IR caused lung vessel congestion (**b**). Staining with H&E. V—vessel; B—bronchiole.

A comparison of bronchioles stained with H&E showed that IR at doses of 2 and 10 Gy did not affect the integrity of the bronchial epithelium (Figure 3a). Analysis of vascular sections revealed the presence of vessel congestion in the lung tissue of 10 Gy IR rats (Figure 3b). The revealed postmortem congestion of vessels may indicate IR-induced changes in endothelial properties.

### 3.3. IR Causes Specific Changes in the Levels of Intercellular Junction Proteins in Lung Tissue

**Figure 4:**
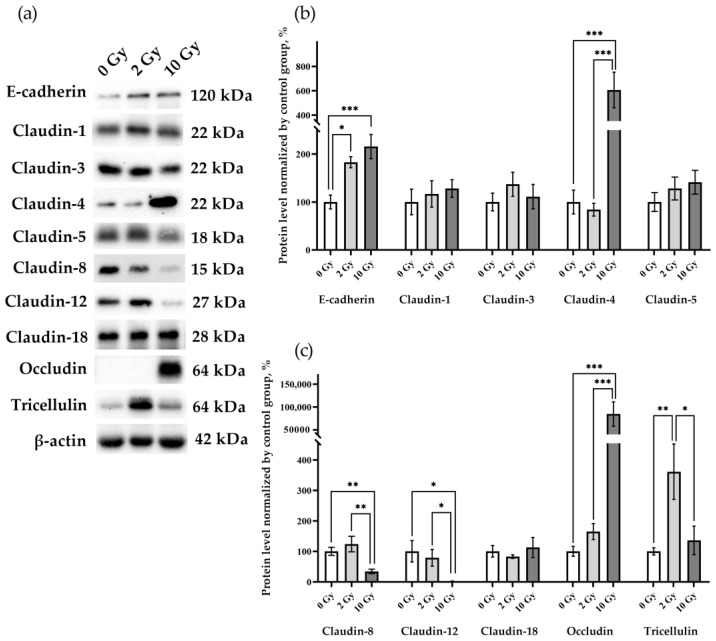
E-cadherin, claudin-1, -3, -4, -5 -8, -12, -18, occludin, and tricellulin in rat lungs 72 h after IR at doses of 0, 2, and 10 Gy. (**a**) Representative blots. Full images of the Western blot are in the Supplementary Material (Figures S2–S12). (**b**,**c**) Western blot analysis of the intercellular junction proteins levels. The intensity of bends was normalized to the intensity of β-actin bends in the corresponding samples. One-way ANOVA followed by Tukey’s multiple comparisons test, * *p* < 0.05, ** *p* < 0.01, *** *p* < 0.001; *n* = 7–8.

The effects of IR at doses of 2 and 10 Gy on the expression of the intercellular junction proteins were analyzed. E-cadherin in the lung tissue of rats receiving 2 Gy and 10 Gy IR was higher than in the sham-irradiated group (Figure 4a,b). The data identified changes in the levels of claudin-4, -8, -12, occludin, and tricellulin, while claudin-1, -3, -5, and -18 were similar in the lung tissue of rats from all studied groups (Figure 4). Claudin-2 was not detected in the lung tissue of rats of the control group or in the lungs of animals after IR at doses of 2 and 10 Gy. Claudin-4 and occludin expression was increased by more than 6- and 80-fold, respectively, in 10 Gy IR rats (Figure 4a,b). The increase in tricellulin was detected only in the lungs of rats treated with 2 Gy IR (Figure 4a,c). Thus, IR causes multidirectional alterations in the levels of intercellular junction proteins in lung tissue.

### 3.4. Evaluation of Processes Induced by IR

**Figure 5:**
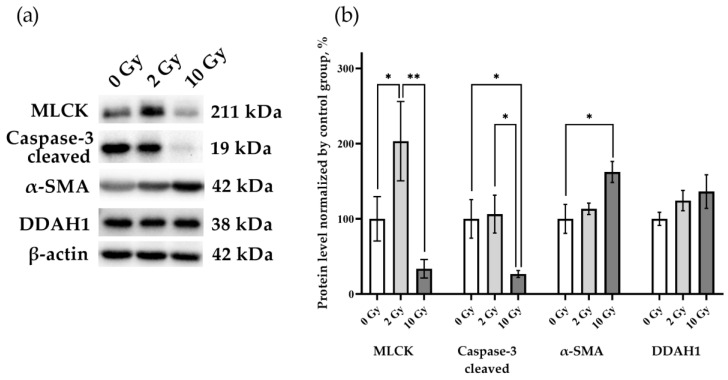
MLCK, cleaved caspase-3, α-SMA, and DDAH1 in rat lungs 72 h after IR at doses of 0, 2, and 10 Gy. (**a**) Representative blots. Full images of the Western blot are in the Supplementary Material (Figures S13–S16). (**b**) Western blot analysis of MLCK, cleaved caspase-3, α-SMA, and DDAH1. The intensity of bends was normalized to the intensity of β-actin bends in the corresponding samples. One-way ANOVA followed by Tukey’s multiple comparisons test, * *p* < 0.05, ** *p* < 0.01; *n* = 7–8.

MLCK, caspase-3, alpha smooth muscle actin (α-SMA), and DDAH1 were assessed to identify the processes occurring in the lung tissue (Figure 5). An increase in MLCK was found in rat lung tissue after IR 2 Gy (*p* < 0.05), indicating that the level of this enzyme is sensitive to the effects of medium doses of IR. A decrease in caspase-3 levels was detected in lung tissue on the third day after 10 Gy IR (*p* < 0.05). Similar expression of DDAH1 in the lung tissue of rats of all studied groups indicates the absence of activation of the DDAH1-iNOS system, which determines the level of nitric oxide in the tissue. Thereby, secondary nitric oxide damage regulated by DDAH1 did not affect the results of our study.

### 3.5. Distribution of Intercellular Junction Proteins in Rat Lung Tissue

**Figure 6:**
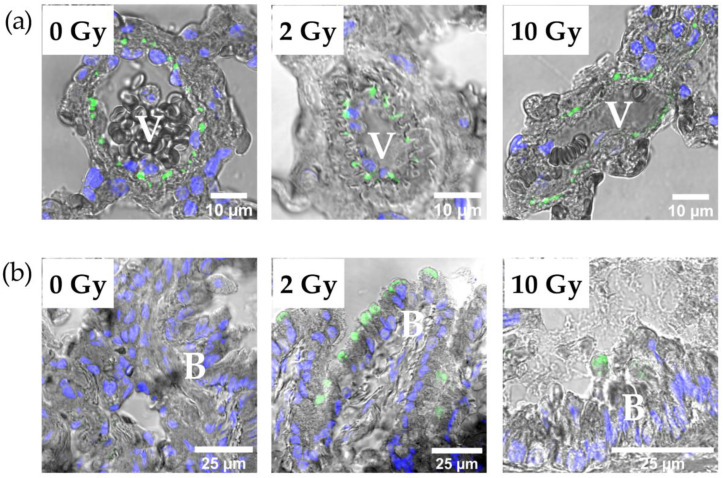
Immunofluorescence staining of lung tissue with antibody to claudin-8 (green) and DAPI (blue). (**a**) Claudin-8 in tight junctions between endothelial cells in rat lungs after IR at doses of 0, 2, and 10 Gy. (**b**) IR at doses of 2 and 10 Gy caused the appearance of claudin-8 in bronchial epithelial cells. V—vessel; B—bronchus.

**Figure 7:**
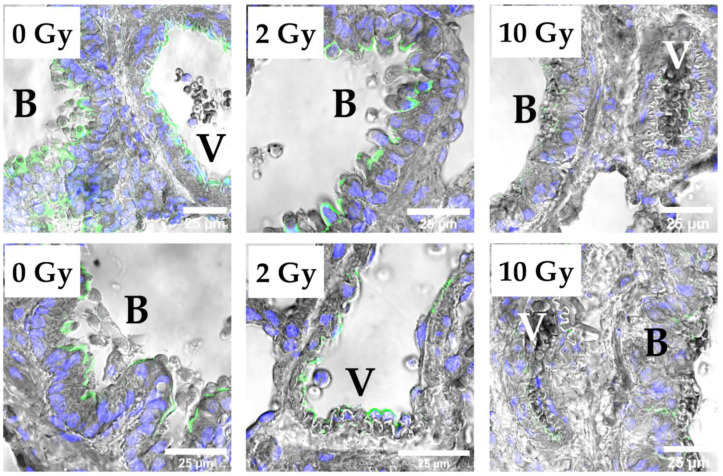
Immunofluorescence staining of lung tissue with antibody to claudin-12 (green) and DAPI (blue). Claudin-12 in bronchial epithelium and endothelium of rat lung tissue of the control group (0 Gy) and 2 Gy IR rats. The 10 Gy IR caused the disappearance of claudin-12 from most of the tight junctions. V—vessel; B—bronchus.

Evaluation of immunofluorescence staining images showed the distribution of the investigated proteins in the lung tissue and the influence of IR on this distribution (Figure 6, Figure 7 and Figures S17–S22). E-cadherin, claudin-1, -3, -4, -18, and occludin were detected in the alveolar epithelium. E-cadherin, claudin-3, claudin-8, and claudin-12 were identified in the intercellular junctions of bronchial epithelium. The distribution pattern of E-cadherin, claudin-1, -3, -4, -18, and occludin was similar in the lung tissue of the control and study groups (Figures S17–S22). Staining with antibodies to claudin-8 showed the presence of this protein in the junctions between endothelial cells (Figure 6a). Image analysis revealed that IR at doses of 2 and 10 Gy induced the appearance of claudin-8 in bronchial epithelium (Figure 6b). Claudin-12 was detected in the lung tissue of rats of the control group in bronchial epithelium and endothelium (Figure 7). The 2 Gy IR did not alter the distribution of claudin-12. In contrast, a 10 Gy dose resulted in the disappearance of claudin-12 from most TJs in bronchial epithelium and endothelium.

## 4. Discussion

Multiprotein complexes of intercellular junction, namely, their constituent proteins, determine intercellular adhesion, permeability, and barrier properties of epithelia. Different diseases and damaging effects lead to changes in the levels of intercellular junction proteins [40,41]. These changes may be adaptive, i.e., contributing to the preservation or restoration of epithelial properties, or desadaptative, leading to epithelial dysfunction and unfavorable consequences for the organism.

The findings demonstrated the increase in E-cadherin on the third day after irradiation at both 2 and 10 Gy doses. This indicates the sensitivity of adherent junctions to IR at a dose of 2 or more Gy. Increased level of E-cadherin is known to suppress EMT processes and promote regeneration of epithelia after injuries [42]. These data allow us to interpret the detected increase in the E-cadherin expression as an adaptive process. It should be noted that the decrease in the level of E-cadherin is typical for later periods after IR exposure [43,44,45,46]. Thereby, a distinctive feature of the first few days after exposure to medium and high doses of IR is an adaptive response of adherent junctions in the form of E-cadherin elevation.

This study is, as far as we know, the first to provide data on changes in TJ protein composition in the lungs after exposure to IR at medium and high doses. Comparison of the effects of medium and high doses of IR on TJ protein levels revealed clear differences between the changes induced by the studied doses. Therefore, 2 Gy IR affected only the tricellulin level, while changes in claudins and occludin were detected only after IR at a dose of 10 Gy.

IR at a dose of 2 Gy in addition to increasing the level of tricellulin in lung tissue induced the appearance of claudin-8 in the bronchial epithelium. It has previously been shown that an increase in claudin-8 levels leads to an increase in epithelial barrier properties [47,48]. These data allow us to suggest that the 2 Gy IR-induced appearance of claudin-8 in rat bronchial epithelium could be seen as an adaptive response.

According to our data, the level of claudin-4 increases in the alveolar epithelium in the first few days after 10 Gy IR. The increase of claudin-4 levels is thought to be a protective response of lung tissue to an acute injury [29,49]. Increasing claudin-4 levels has the effect of enhancing alveolar fluid clearance, which is manifested by preventing alveolar filling after injury [50]. An in vitro study has shown that an elevation of claudin-4 levels in alveolar epithelial cells leads to an increase in the barrier properties of the epithelium [51]. Thus, the increase in the claudin-4 level at the early stages after 10 Gy IR is probably a sign of an adaptive response of the alveolar epithelium.

It has been shown that in addition to affecting epithelial barrier properties, the elevation of claudin-4 levels also leads to an inhibition of apoptosis [34]. We revealed both a decline in activated caspase-3 levels and increase in claudin-4 levels, which is in line with previously published data [34]. Thus, the next possible adaptive effect observed in lung tissue on the third day after 10 Gy IR is claudin-4-dependent inhibition of apoptosis.

Bronchial epithelium had decreased level of claudin-12 and appearance of claudin-8 after 10 Gy IR. The same exposure resulted in a reduction in both claudin-8 and claudin-12 levels in the endothelium. It was mentioned above that the appearance of claudin-8 probably contributes to an increase in the barrier properties of the bronchial epithelium [47,48] Considering the findings of Sun et al.’s [48] study, it can be assumed that the decrease in the level of claudin-8 in endothelium contributes to the increase in vascular permeability. The effect of claudin-12 alterations on the properties of bronchial epithelium and endothelium in lung tissue remains to be elucidated.

One of the most intriguing results was the finding of a catastrophic increase in occludin level in lung tissue on the third day after 10 Gy IR. These findings are consistent with the results of a study showing an increase in occludin expression in an alveolar epithelial cell line after IR exposure [52]. It is known that in response to various injuries there is a decrease in the level of occludin in lung tissue, and the effectiveness of therapeutic actions is assessed by the restoration of the level of this protein in the lungs [53,54,55,56,57]. Considering these data, it can be assumed that the increase in occludin level can be regarded as an adaptation process in lung tissue.

Thus, the results of the study revealed that in the first days after IR in medium and high doses there are changes in the structure of lung tissue, as well as multidirectional alterations in the levels and distribution of intercellular junction proteins. Furthermore, at least part of the identified changes (increased levels of E-cadherin, claudin-4, and occludin) is associated with adaptive processes. The findings indicate the prospect of further investigations of early changes in lung tissue after IR.

## Data Availability

The data that support the findings of this study are available from the corresponding author upon reasonable request.

## Supplementary Materials

The following supporting information can be downloaded at: https://www.mdpi.com/article/10.3390/pathophysiology31040039/s1, Figure S1. Scheme of stages of lung tissue slice images preparation for morphometry. Figure S2. E-cadherin in rat lungs 72 h after IR at doses of 0, 2, and 10 Gy. Results of Western blot. Figure S3. Claudin-1 in rat lungs 72 h after IR at doses of 0, 2, and 10 Gy. Results of Western blot. Figure S4. Absence of claudin-2 in rat lungs 72 h after irradiation at doses of 0, 2, and 10 Gy. Western blot results. Exposure 12 min. Figure S5. Claudin-3 in rat lungs 72 h after IR at doses of 0, 2, and 10 Gy. Results of Western blot. Figure S6. Claudin-4 in rat lungs 72 h after IR at doses of 0, 2, and 10 Gy. Results of Western blot. Figure S7. Claudin-5 in rat lungs 72 h after IR at doses of 0, 2, and 10 Gy. Results of Western blot. Figure S8. Claudin-8 in rat lungs 72 h after IR at doses of 0, 2, and 10 Gy. Results of Western blot. Figure S9. Claudin-12 in rat lungs 72 h after IR at doses of 0, 2, and 10 Gy. Results of Western blot. Figure S10. Claudin-18 in rat lungs 72 h after IR at doses of 0, 2, and 10 Gy. Results of Western blot. Figure S11. Occludin in rat lungs 72 h after IR at doses of 0, 2, and 10 Gy. Results of Western blot. Figure S12. Tricellulin in rat lungs 72 h after IR at doses of 0, 2, and 10 Gy. Results of Western blot. Figure S13. MLCK in rat lungs 72 h after IR at doses of 0, 2, and 10 Gy. Results of Western blot. Figure S14. Cleaved caspase-3 in rat lungs 72 h after IR at doses of 0, 2, and 10 Gy. Results of Western blot. Figure S15. Alpha-smooth muscle actin in rat lungs 72 h after IR at doses of 0, 2, and 10 Gy. Results of Western blot. Figure S16. DDAH1 in rat lungs 72 h after IR at doses of 0, 2, and 10 Gy. Results of Western blot. Figure S17. Immunofluorescence staining of lung tissue with antibody to E-cadherin (green) and DAPI (blue). E-cadherin in tight junctions between alveolar epithelial cells in rat lungs after IR at doses of 0, 2, and 10 Gy. Figure S18. Immunofluorescence staining of lung tissue with antibody to claudin-1 (green) and DAPI (blue). Claudin-1 in tight junctions between alveolar epithelial cells in rat lungs after IR at doses of 0, 2, and 10 Gy. Figure S19. Immunofluorescence staining of lung tissue with antibody to claudin-3 (green) and DAPI (blue). Claudin-3 in tight junctions between bronchial epithelial cells and alveolar epithelial cells in rat lungs after IR at doses of 0, 2, and 10 Gy. Figure S20. Immunofluorescence staining of lung tissue with antibody to claudin-4 (green) and DAPI (blue). Claudin-4 in tight junctions between alveolar epithelial cells in rat lungs after IR at doses of 0, 2, and 10 Gy. Figure S21. Immunofluorescence staining of lung tissue with antibody to claudin-18 (green) and DAPI (blue). Claudin-18 in tight junctions between alveolar epithelial cells in rat lungs after IR at doses of 0, 2, and 10 Gy. Figure S22. Immunofluorescence staining of lung tissue with antibody to occludin (green) and DAPI (blue). Occludin in tight junctions between alveolar epithelial cells in rat lungs after IR at doses of 0, 2, and 10 Gy.
