# Supplementary material for: Early Radiation-Induced Changes in Lung Tissue and Intercellular Junctions: Implications for Tissue Repair and Fibrosis
Source: Pathophysiology. 2024 Sep 24;31(4):531–44. doi: 10.3390/pathophysiology31040039 (PMC11503413; doi:10.3390/pathophysiology31040039)
Supplement: Supplementary file 1 [file pathophysiology-31-00039-s001.zip › pathophysiology-3117930-supplementary.pdf]

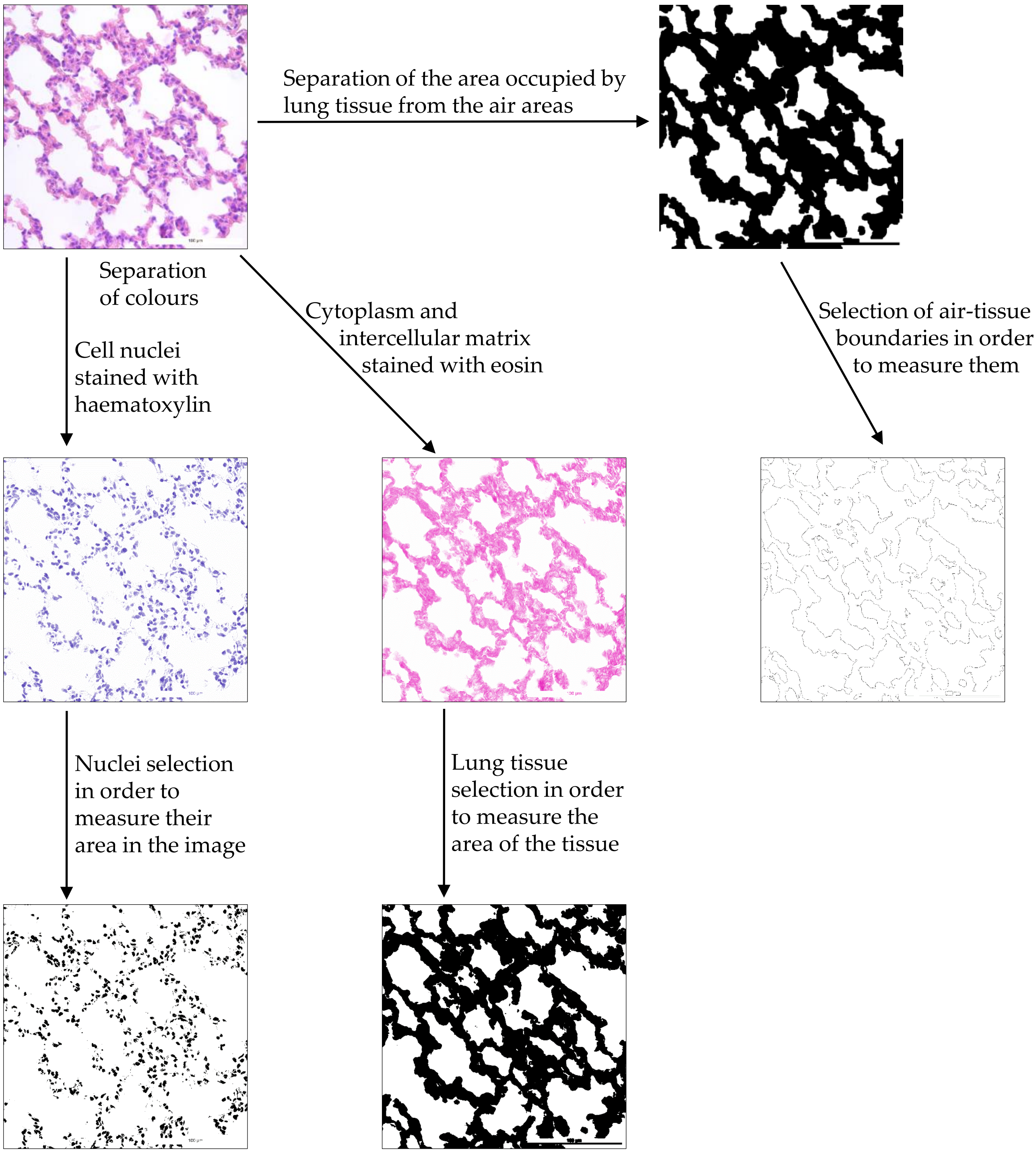

Figure S1. Scheme of stages of lung tissue slice images preparation for morphometry.

E-cadherin

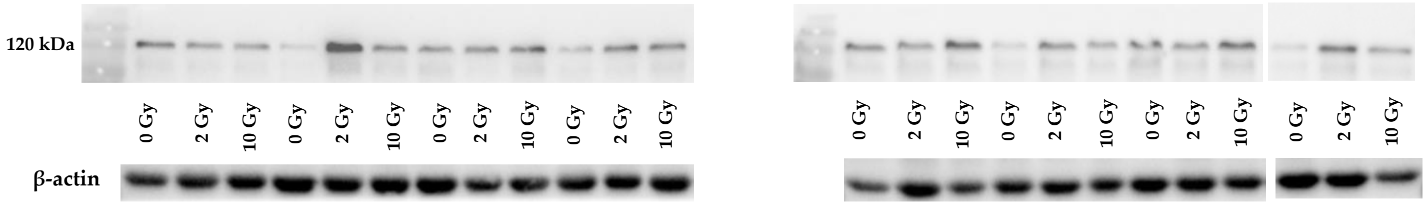

Figure S2. E-cadherin in rat lungs 72 hours after IR at doses of 0, 2 and 10 Gy. Results of Western blot.

Claudin-1

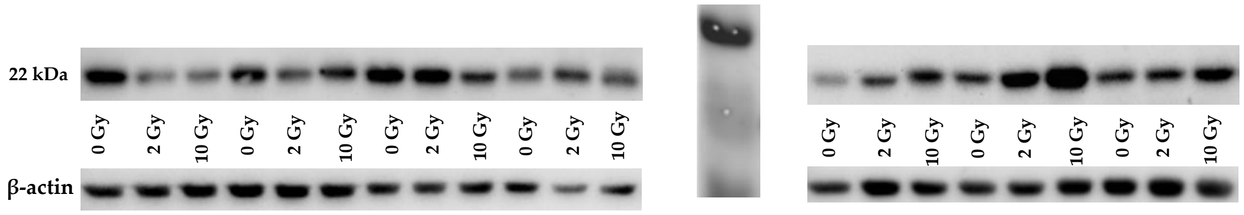

Figure S3. Claudin-1 in rat lungs 72 hours after IR at doses of 0, 2 and 10 Gy. Results of Western blot.

Claudin-2

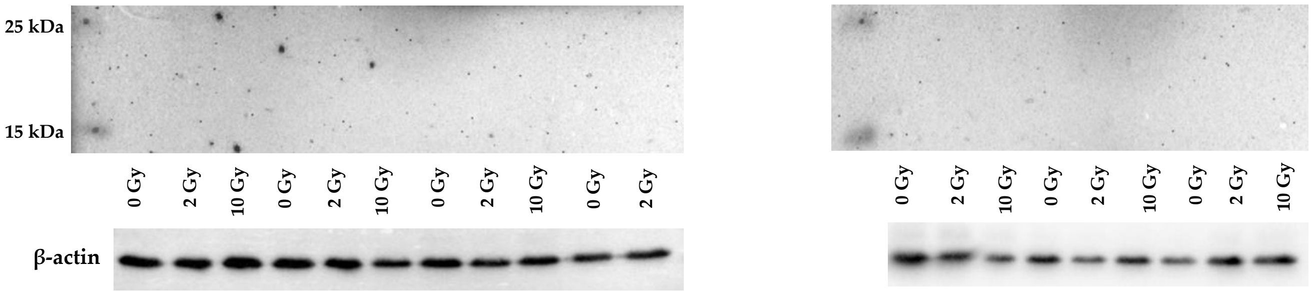

Figure S4. Absence of claudin-2 in rat lungs 72 hours after irradiation at doses of 0, 2 and 10 Gy. Western blot results. Exposure 12 minutes.

Claudin-3

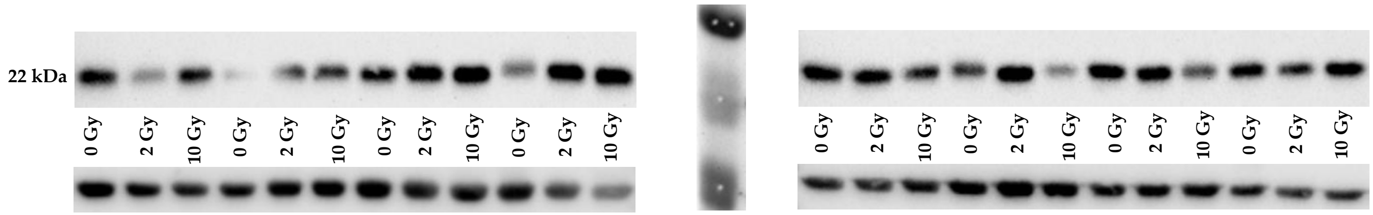

Figure S5. Claudin-3 in rat lungs 72 hours after IR at doses of 0, 2 and 10 Gy. Results of Western blot.

Claudin-4

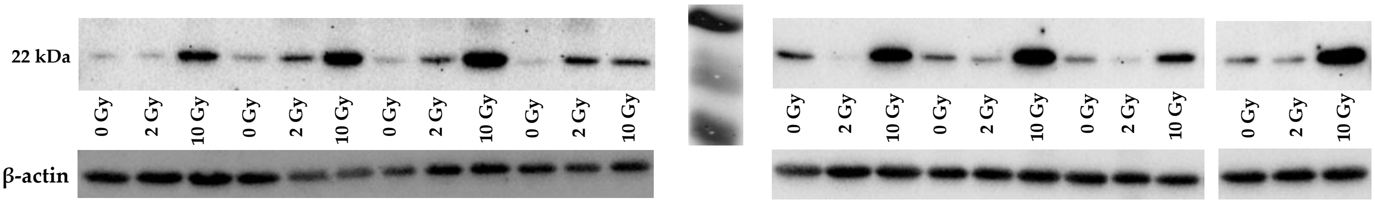

Figure S6. Claudin-4 in rat lungs 72 hours after IR at doses of 0, 2 and 10 Gy. Results of Western blot.

Claudin-5

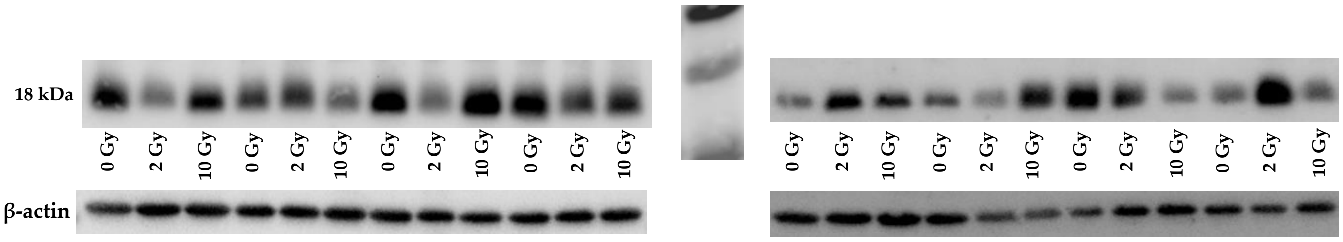

Figure S7. Claudin-5 in rat lungs 72 hours after IR at doses of 0, 2 and 10 Gy. Results of Western blot.

Claudin-8

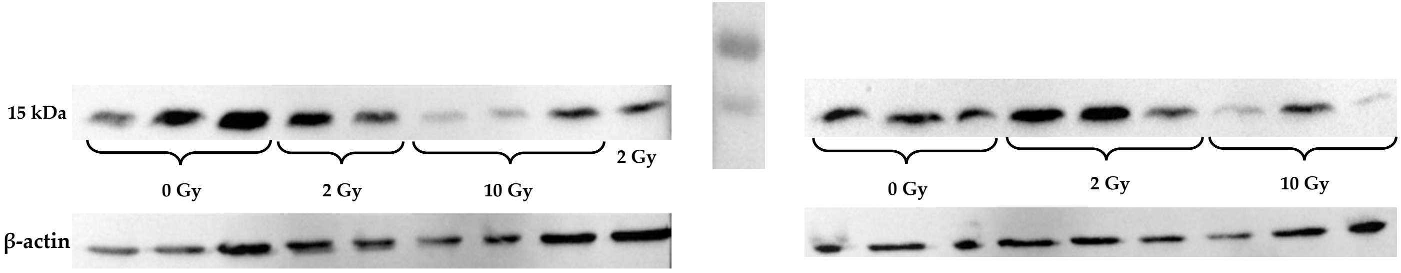

Figure S8. Claudin-8 in rat lungs 72 hours after IR at doses of 0, 2 and 10 Gy. Results of Western blot.

Claudin-12

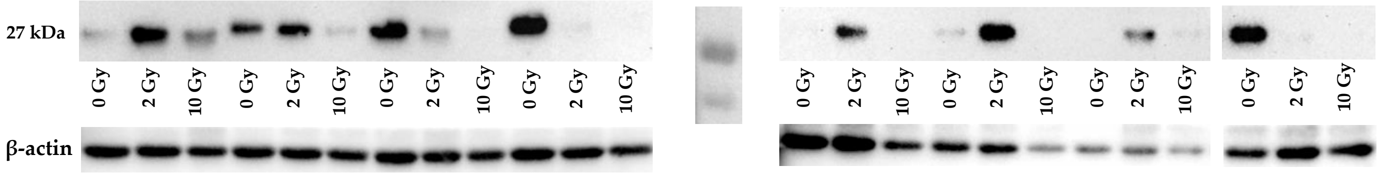

Figure S9. Claudin-12 in rat lungs 72 hours after IR at doses of 0, 2 and 10 Gy. Results of Western blot.

Claudin-18

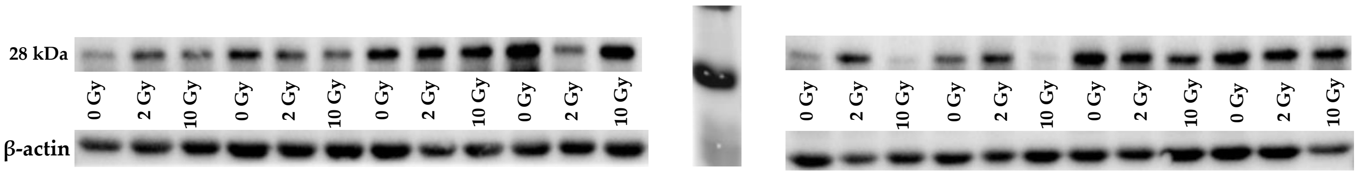

Figure S10. Claudin-18 in rat lungs 72 hours after IR at doses of 0, 2 and 10 Gy. Results of Western blot.

Occludin

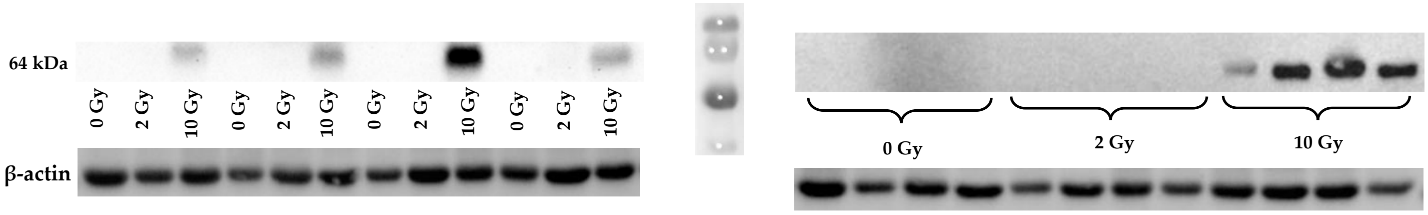

Figure S11. Occludin in rat lungs 72 hours after IR at doses of 0, 2 and 10 Gy. Results of Western blot.

Tricellulin

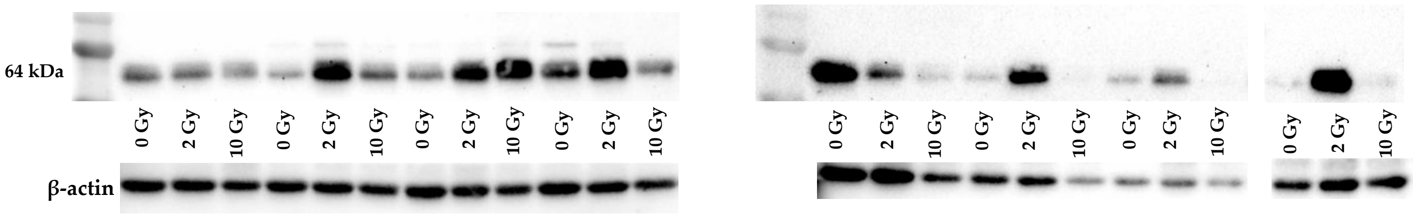

Figure S12. Tricellulin in rat lungs 72 hours after IR at doses of 0, 2 and 10 Gy. Results of Western blot.

MLCK

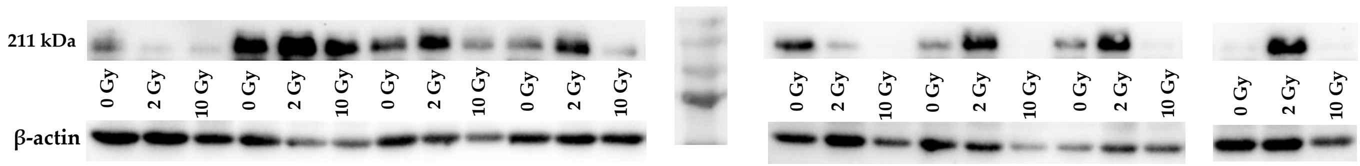

Figure S13. MLCK in rat lungs 72 hours after IR at doses of 0, 2 and 10 Gy. Results of Western blot.

Caspase-3 cleaved

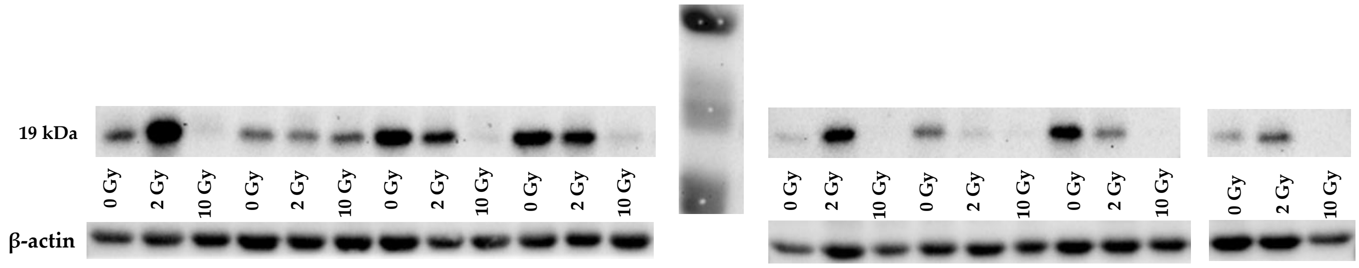

Figure S14. Cleaved caspase-3 in rat lungs 72 hours after IR at doses of 0, 2 and 10 Gy. Results of Western blot.

Alpha-smooth muscle actin

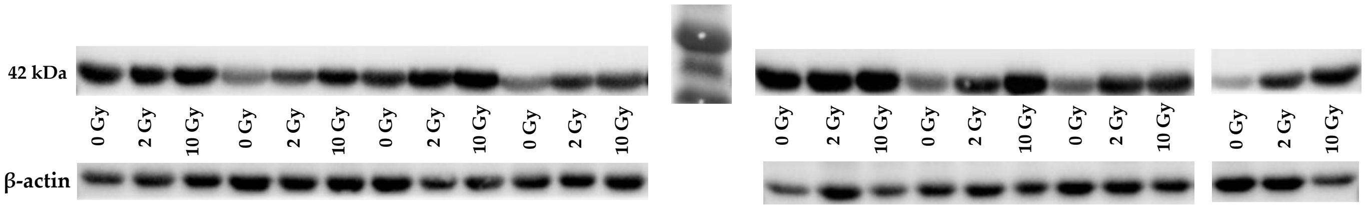

Figure S15. Alpha-smooth muscle actin in rat lungs 72 hours after IR at doses of 0, 2 and 10 Gy. Results of Western blot.

DDAH1

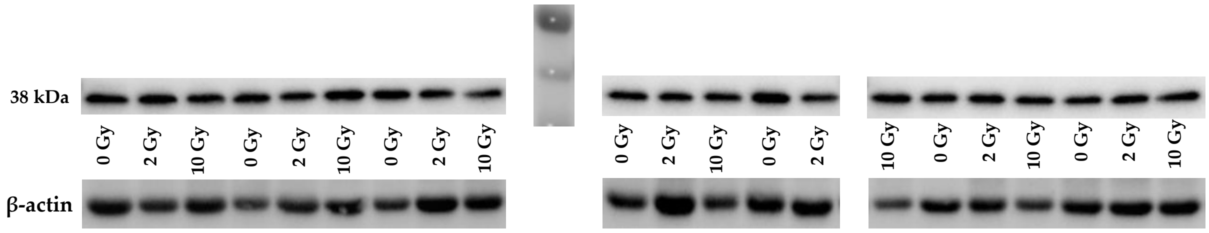

Figure S16. DDAH1 in rat lungs 72 hours after IR at doses of 0, 2 and 10 Gy. Results of Western blot.

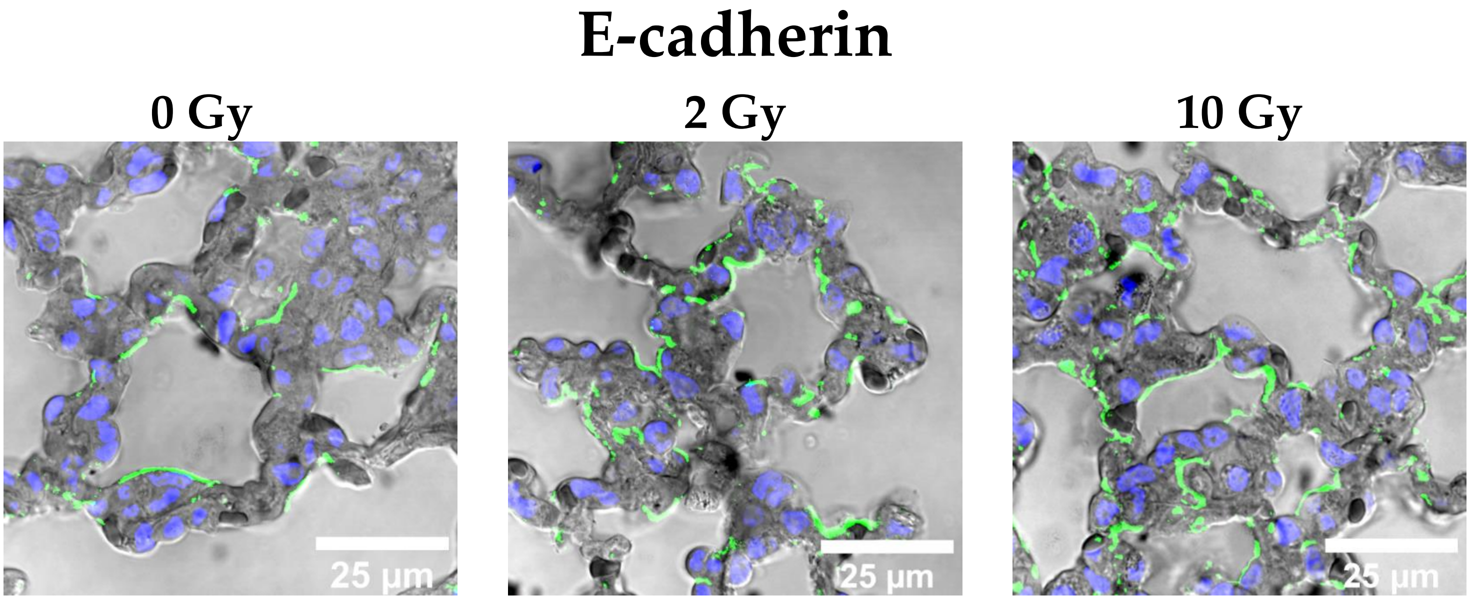

Figure S17. Immunofluorescence staining of lung tissue with antibody to E-cadherin (green) and DAPI (blue). E-cadherin in tight junctions between alveolar epithelial cells in rat lungs after IR at doses of 0, 2 and 10 Gy.

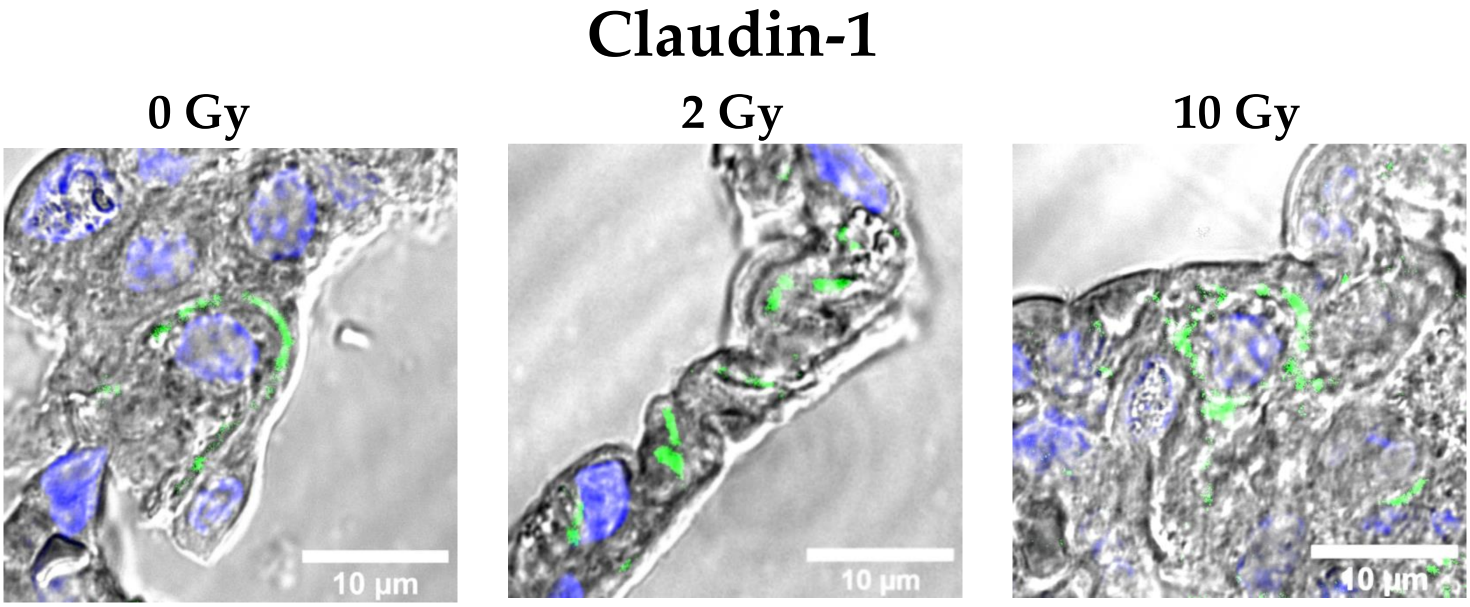

Figure S18. Immunofluorescence staining of lung tissue with antibody to claudin-1 (green) and DAPI (blue). Claudin-1 in tight junctions between alveolar epithelial cells in rat lungs after IR at doses of 0, 2 and 10 Gy.

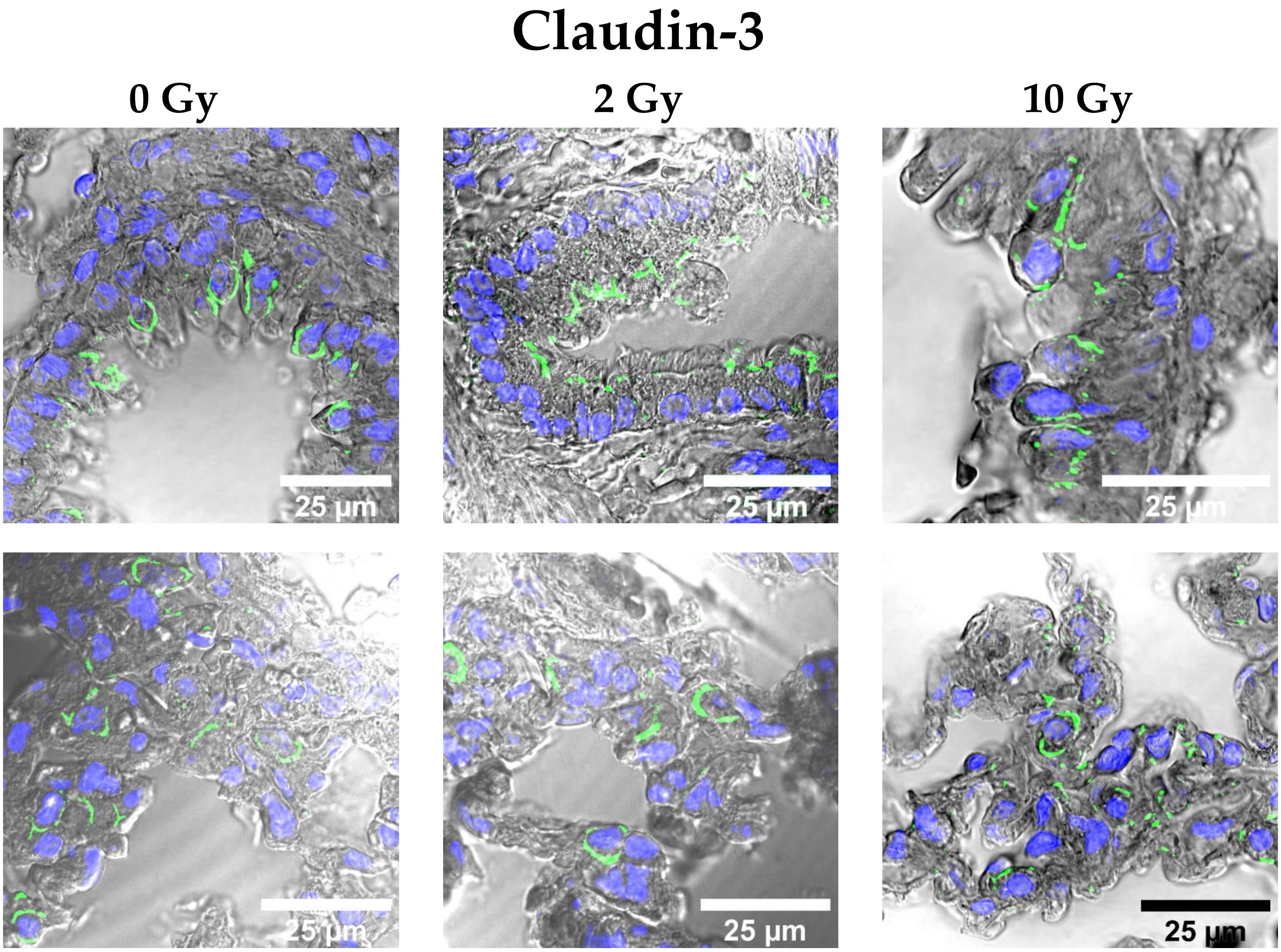

Figure S19. Immunofluorescence staining of lung tissue with antibody to claudin-3 (green) and DAPI (blue). Claudin-3 in tight junctions between bronchial epithelial cells and alveolar epithelial cells in rat lungs after IR at doses of 0, 2 and 10 Gy.

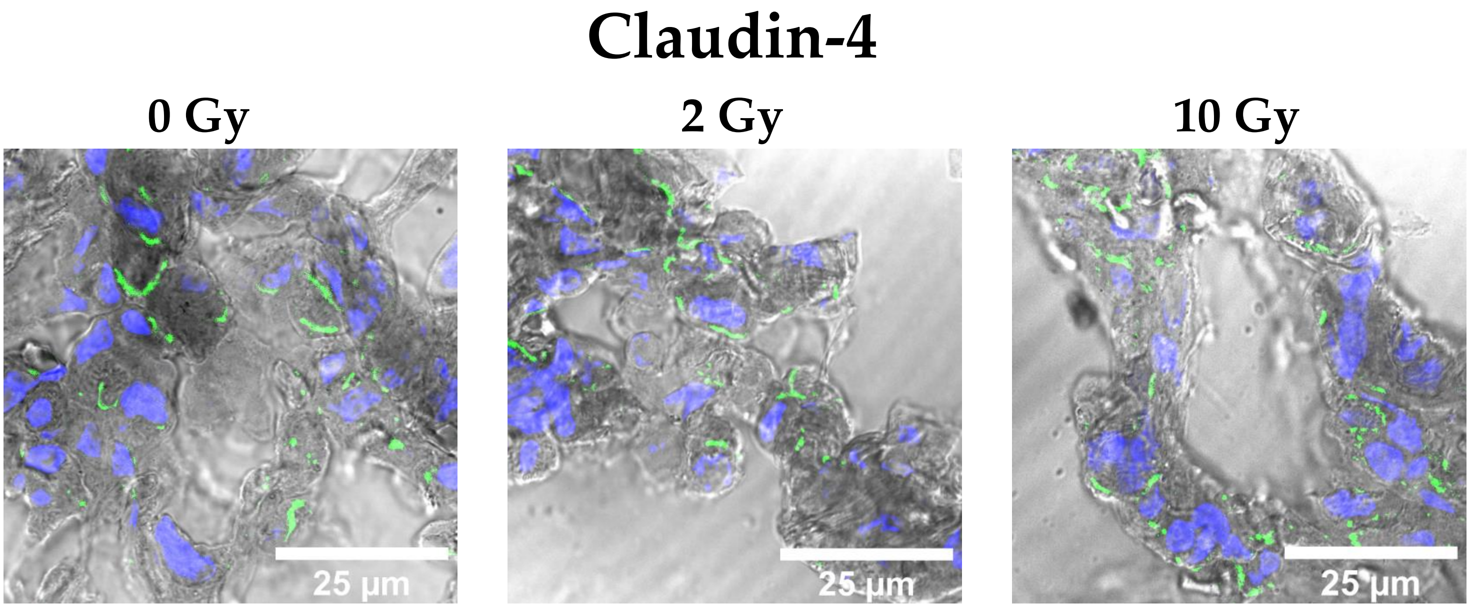

Figure S20. Immunofluorescence staining of lung tissue with antibody to claudin-4 (green) and DAPI (blue). Claudin-4 in tight junctions between alveolar epithelial cells in rat lungs after IR at doses of 0, 2 and 10 Gy.

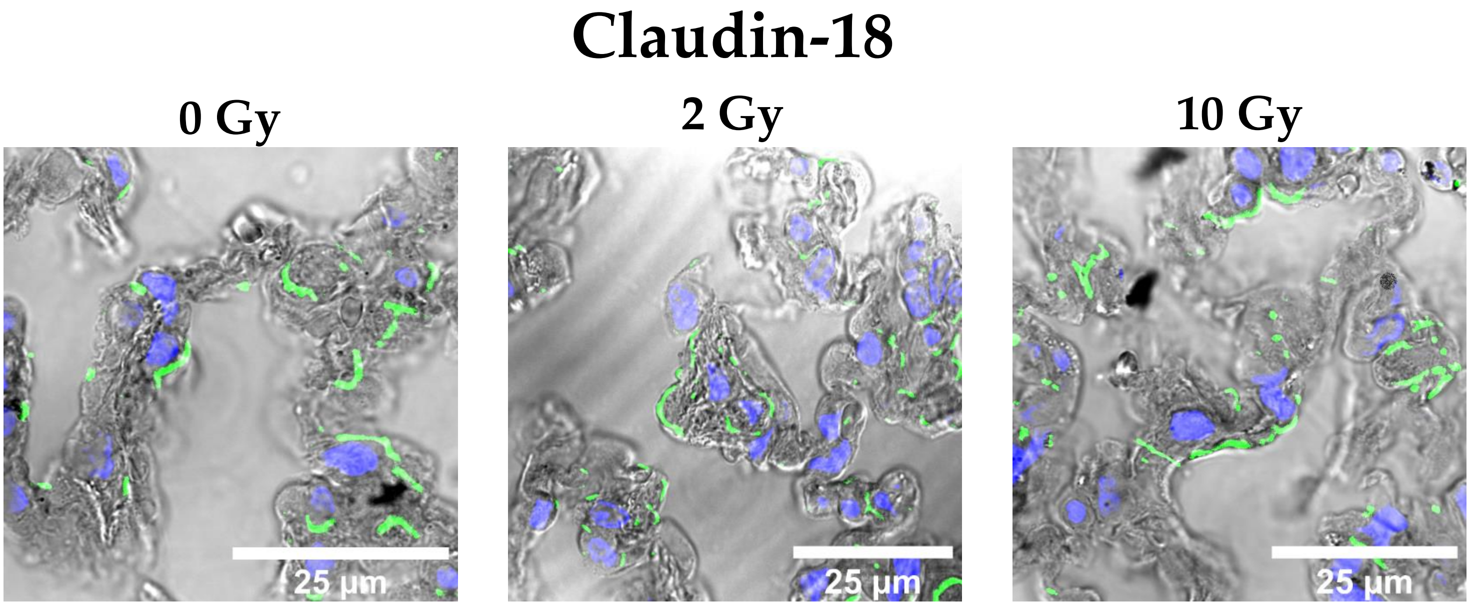

Figure S21. Immunofluorescence staining of lung tissue with antibody to claudin-18 (green) and DAPI (blue). Claudin-18 in tight junctions between alveolar epithelial cells in rat lungs after IR at doses of 0, 2 and 10 Gy.

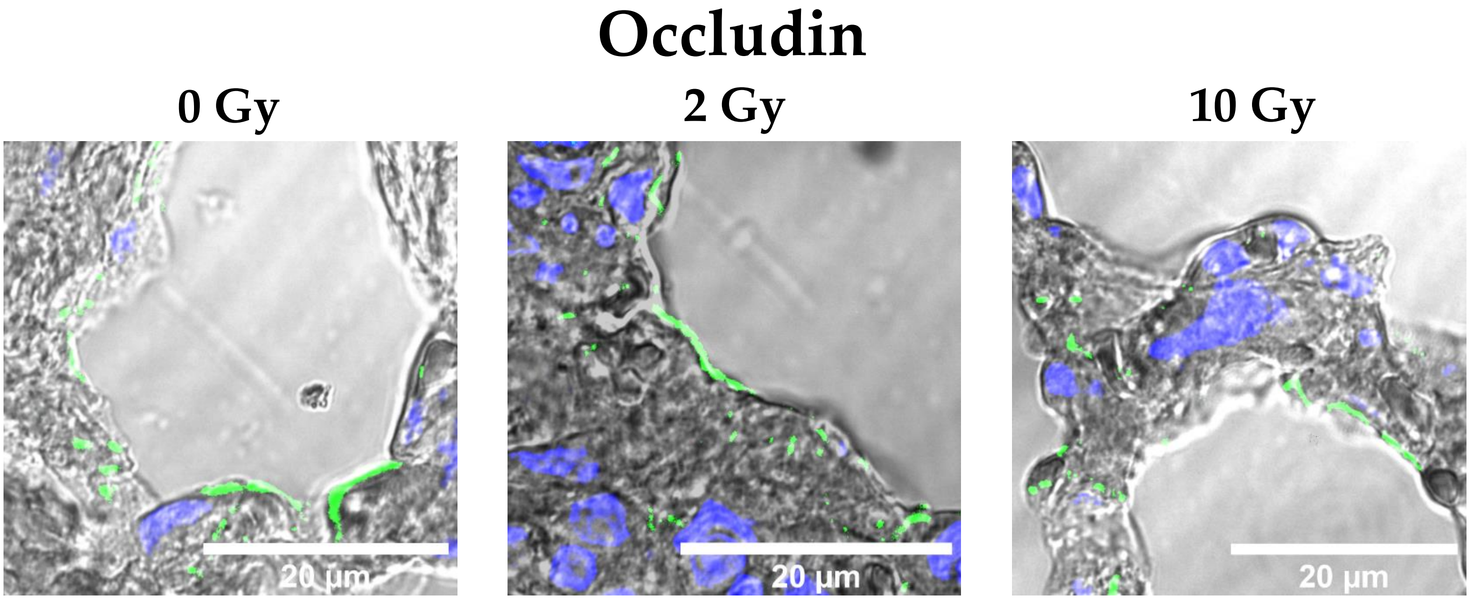

Figure S22. Immunofluorescence staining of lung tissue with antibody to occludin (green) and DAPI (blue). Occludin in tight junctions between alveolar epithelial cells in rat lungs after IR at doses of 0, 2 and 10 Gy.
